# Risk factors for severe COVID-19 disease increase SARS-CoV-2 infectivity of endothelial cells and pericytes

**DOI:** 10.1098/rsob.230349

**Published:** 2024-06-12

**Authors:** Luca Biasetti, Nikos Zervogiannis, Kira Shaw, Harry Trewhitt, Louise Serpell, Dalan Bailey, Edward Wright, Catherine N. Hall

**Affiliations:** ^1^ Sussex Neuroscience, School of Psychology, University of Sussex, East Sussex BN1 9QG, UK; ^2^ Sussex Neuroscience, School of Life Sciences, University of Sussex, East Sussex BN1 9QG, UK; ^3^ The Pirbright Institute, Surrey GU24 0NF, UK; ^4^ Viral Pseudotype Unit, School of Life Sciences, University of Sussex, , East Sussex BN1 9QG, UK

**Keywords:** COVID-19, pericyte, endothelial cell, infection, APOE, SARS-CoV-2

## Abstract

Coronavirus disease 2019 (COVID-19) was initially considered a primarily respiratory disease but is now known to affect other organs including the heart and brain. A major route by which COVID-19 impacts different organs is via the vascular system. We studied the impact of apolipoprotein E (APOE) genotype and inflammation on vascular infectivity by pseudo-typed severe acute respiratory syndrome coronavirus 2 (SARS-CoV-2) viruses in mouse and human cultured endothelial cells and pericytes. Possessing the APOE4 allele or having existing systemic inflammation is known to enhance the severity of COVID-19. Using targeted replacement human APOE3 and APOE4 mice and inflammation induced by bacterial lipopolysaccharide (LPS), we investigated infection by SARS-CoV-2. Here, we show that infectivity was higher in murine cerebrovascular pericytes compared to endothelial cells and higher in cultures expressing APOE4. Furthermore, increasing the inflammatory state of the cells by prior incubation with LPS increased infectivity into human and mouse pericytes and human endothelial cells. Our findings provide insights into the mechanisms underlying severe COVID-19 infection, highlighting how risk factors such as APOE4 genotype and prior inflammation may exacerbate disease severity by augmenting the virus’s ability to infect vascular cells.

## 1. Introduction

Coronavirus disease 2019 (COVID-19), caused by the severe acute respiratory syndrome coronavirus 2 (SARS-CoV-2), is estimated to have caused over 620 billion infections globally and over 6 million deaths (World Health Organization). COVID-19 does not just cause acute disease, as 1 in 5 of survivors have symptoms that last over five weeks. Moreover, being hospitalized with COVID-19 increases the risk of subsequently suffering from cardiovascular, kidney or lung diseases, or of developing diabetes [1–3]. As most patients experience initial pulmonary symptoms, SARS-CoV-2 was initially considered primarily a respiratory disease; however, acute and chronic (or long) COVID-19 also affects many other organs, for example, producing cardiac dysfunction, dysautonomia and cognitive dysfunction [4–7]. The route by which COVID-19 impacts different organs is increasingly appreciated to be via the vascular system: SARS-CoV-2 infection of vascular cells produces thrombosis, endothelial dysfunction and degeneration, as well as dysregulated angiogenesis [8–10]. Vascular risk factors predispose patients to suffering from more severe COVID-19 [11], and conversely, the severity of COVID-19 in patients has been linked to the degree to which endothelial dysfunction occurs [12]. Post-acute (long) COVID-19 symptoms have also been associated with ongoing vascular dysfunction [13–16]. Parenchymal tissue may then be adversely affected by the lack of blood supply, the release of cytotoxic molecules, increased vascular leakiness or by invasion of the virus itself and infection of parenchymal cells [17]. Because of the importance of the vasculature in driving acute and chronic disease pathology, it will be vital to understand the factors that affect vascular infection with SARS-CoV-2 for targeting COVID-19 interventions and understanding its short- and long-term effects.

Despite the clear importance of vascular infection in COVID-19 disease, it remains unclear which vascular cells are targeted by SARS-CoV-2. The major route for cells to become infected is via the spike (S) protein on SARS-CoV-2 virions binding to angiotensin-converting enzyme 2 (ACE2) and being cleaved by the transmembrane serine protease TMPRSS2 [18] or cathepsins B or L [19,20], and also possibly furin [21]. ACE2 is expressed in pericytes, which are vascular mural cells that exist abluminally to endothelial cells, meaning pericytes can become infected with SARS-CoV-2 [22–24]. However, whether endothelial cells, which are in direct contact with the blood vessel lumen, can be infected with SARS-CoV-2 remains controversial [25]. Some studies propose that endothelial cells do express ACE2 [26], while others argue that endothelial cell expression of ACE2 is low to absent at both the protein and mRNA level [12,27]. Entry may also occur via ACE2-independent routes such as binding to transmembrane protein 106B (TMEM106B) [28], which may be expressed in endothelial cells and pericytes [23,29]. Evidence of viral particle inclusion in endothelial cells is also mixed [25], although the effects of SARS-CoV-2 proteins on endothelial pathology have been reported [27].

With such variability in the symptoms and severity observed in patients suffering from acute and long COVID-19 and such mixed results surrounding levels of infection of vascular cells, we hypothesized that vascular infectivity could be modulated by factors that increase disease severity. We, therefore, studied the impact of two factors known to increase severity of COVID-19 disease on vascular infectivity. We studied permissiveness of cultured endothelial cells and pericytes from mouse and human to pseudo-typed SARS-CoV-2. These viruses bear the S protein from different variants of concern (VOC) but cannot replicate in the cell, so are ideal for studying the site of first infection in the vascular system. First, we studied the impact of apolipoprotein E (APOE) genotype on endothelial and pericyte infectivity. Humans carry APOE2, APOE3 or APOE4 alleles of the *APOE* gene. Carrying the APOE4 allele increases the severity of COVID-19 disease in patients [30] and increases neuronal and astrocytic infection with SARS-CoV-2 [31], as well as impairing vascular function [32]. Second, we used application of bacterial lipopolysaccharide (LPS) to mimic existing inflammation, which is a common link between multiple risk factors for severe COVID-19, including diabetes, hypertension and obesity [33]. Infectivity of endothelial cells and pericytes was increased by both APOE4 genotype and by previous exposure to LPS. We also observed important differences across the COVID-19 VOC. Therefore, risk factors for severe COVID-19 may mediate their effects, at least in part, by modulating the potential for SARS-CoV-2 to infect vascular cells.

## 2. Methods

### (a) Animals

For the preparation of dissociated mouse endothelial and pericyte cultures, mice homozygous for the targeted replacement (TR) of the murine *APOE* gene with either the human *APOE4* (*n* = 12, six males and six females, 9–12 weeks old) or *APOE3* genes (*n* = 13, nine males and four females, 7–11 weeks old; derived from founders provided by N. Maeda (UNC School of Medicine, USA) were used. For each individual culture preparation, usually, a total of three brains were used, of mixed sex. In some instances, only two brains were used, and the volumes of solutions and plating areas were adapted accordingly. Different experimental runs were performed on independent cultures.

### (b) Cell culture

#### (i) Dissociated mouse endothelial and pericyte cultures preparation

The protocol used for the preparation of primary endothelial and pericyte mouse cultures was reported by Boroujerdi *et al.* [34] and Mehra *et al.* [35]. Briefly, two wells of a 6-well plate were coated with 2 ml per well of collagen coating solution (1 : 5 dilution from stock collagen type 1 in sterile water; see table 1 for sources of general reagents) for 2 h at 37°C in 5% CO_2_. Following three washes with phosphate-buffered saline (PBS 1×; 0.01 M phosphate buffer, 0.0027 M potassium chloride and 0.137 M sodium chloride (pH 7.4) at 25°C), the plates were left in in the incubator to allow for gas equilibration before plating. Three brains from 7- to 12-week-old mice were harvested following cervical dislocation, and the tissue was submerged in an ice-cold dissecting solution (table 2) in a small Petri dish. The meninges were carefully removed, together with the cerebellum, striatum and olfactory bulbs. Each individual brain was then cut sagittally, and both hemispheres were transferred to a Dounce tissue grinder to be homogenized together with 2 ml of dissecting solution. The tissue was minced 55 times with a ‘loose’ pestle, followed by 25 times with a ‘tight’ pestle; before the resulting slurry from the three brains was combined and centrifuged at 180*g* for 5 min. The supernatant was then removed, and the resuspended pellet was incubated in a papain (30 U ml^−1^)/DNAse I solution (40 μg ml^−1^) at 37°C for 1 h and 10 min. Following this step, 3.5 ml of bovine serum albumin (BSA, 22%) was added to the brain homogenate, and the sample was once again centrifuged, this time at 390*g* for 10 min. The supernatant, now containing myelin and BSA, was then removed, and the pellet containing vascular tubes was resuspended in 1 ml endothelial cell growth medium (ECGM; table 3), before transferring to a new 15 ml tube, adding another 1 ml of media and spinning it down at 180*g* for 5 min. The washed blood vessels were resuspended in 4 ml of ECGM and plated onto the collagen-treated plates, 2 ml per well, and incubated overnight. The following morning, a full media change was performed and cells were left to grow to confluence. When cells had reached confluence, cultures were passaged. Briefly, four wells of a new 6-well plate were coated in collagen for 2 h at 37°C, before undergoing three washes in PBS (1×) and then kept in the incubator to equilibrate. ECGM was removed from the confluent wells, and cells were washed with F12 media before 2 ml of trypsin was added to each well, and the plate was left in the incubator for 5 min. An additional 2 ml of F12 media was added to each well, and the detached cells were transferred to a 15 ml tube, before being spun down at 180*g* for 5 min. The pellet was resuspended in 1 ml of ECGM, and an additional 3 ml of the same media was then added. At this point, 1 ml of this cell suspension was seeded in each of the previously collagenized wells, where 1 ml of media had already been added and equilibrated at 37°C. When cells reached confluency once more, cultures were passaged again following the same passaging steps just described. The only difference was the plating in pericyte cell growth medium (PCGM; table 4) instead of ECGM, and all passages from here onwards were performed with PCGM. As indicated by Boroujerdi *et al*. [34], while in the early passages, the cell population is predominantly composed of brain endothelial cells, by the fourth passage the cultures become more and more dominated by pericytes, as can be observed by the change in morphology (see the electronic supplementary material, figure S1).

**Table 1.** Sources of general reagents.

materials	product code
collagen type I	Sigma (C3867)
papain	Worthington Biochemical Corp.
DNAse I	Worthington Biochemical Corp.
BSA (30%)	Sigma (A9576)
trypsin	Sigma (T4049)
Ham’s F-12 nutrient mix	Sigma (N6658)

**Table 2.** Dissecting solution components.

materials	product code	volume
Hank’s balanced salt solution	Sigma (H8254)	49 ml
HEPES buffer solution	Sigma (H0887)	0.5 ml
penicillin–streptomycin	Sigma (P0781)	0.5 ml

**Table 3.** Endothelial cell growth medium (ECGM) components.

materials	product code	volume
Gibco Dulbecco′s modified Eagle′s medium/nutrient mixture F-12	Sigma (D6421)	48 ml
foetal bovine serum (FBS)	Sigma (F7524)	5 ml
penicillin–streptomycin	Sigma (P0781)	0.5 ml
endothelial cell growth supplement	Sigma (02-102)	100 μl

**Table 4.** Pericyte cell growth medium (PCGM) components.

materials	product code	volume
pericyte (basal) medium	ScienCell (1201)	500 ml
FBS	ScienCell (0010)	10 ml
penicillin–streptomycin	ScienCell (0503)	5 ml
pericyte growth supplement	ScienCell (1252)	5 ml

#### (ii) Human pericyte and endothelial cell cultures

Human endothelial and pericyte cultures were prepared from isolated primary cells. Specifically, endothelial cultures were initially plated from primary human cardiac microvascular endothelial cells isolated from heart ventricles from a single donor (PromoCell, c-12281), while pericyte cultures were prepared from human pericytes isolated from placental tissue (PromoCell, c-12980). Isolated primary cells were received either fresh or cryopreserved. In both cases, cells were initially plated in a T-25 (Thermo Fisher Scientific, 156340) before passaging them onto the preferred plate or flask. Human pericytes were cultured using pericyte growth medium 2 (PromoCell, c-28041; includes basal media and supplement mix), while human endothelial cells were cultured using ECGM MV (PromoCell, c-22020; includes basal media and supplement mix; final supplement concentration is as follows: foetal calf serum 50 μl ml^−1^, endothelial grown supplement 4 μl ml^−1^, endothelial growth factor (recombinant human) 10 ng ml^-1^, heparin 90 μg ml^−1^ and hydrocortisone 1 μg ml^−1^).

#### (iii) Preparation of SARS-Cov-2 pseudo-typed virus

Production of pseudo-typed virus (PV) was carried out via co-transfection of the necessary plasmids into human embryonic kidney (HEK) 293T/17 cells (ATCC, CRL-11268; maintained in high glucose Dulbecco’s modified Eagle’s medium and 10% foetal bovine serum (FBS) with 5% CO_2_). Cells were plated on day 1 in a 10 cm dish at a seeding ratio calculated to deliver a 70%–90% optimal confluency by the time of transfection (on day 2). Briefly, 175 μl of pre-warmed Opti-MEM (Gibco) were added to a 1.5 ml microcentrifuge tube together with the following plasmids: pCAGGS SARS-CoV-2 VOC S (1 μg), p8.91-lentiviral vector (1 μg), pCSFLW (pHR-SIN-CFLW, 1.5 μg), as well as 10.5 μl of FugeneHD [36]. The mixture was left to incubate at room temperature for 10–15 min while the cell media was changed. The mixture was then added dropwise to the cells, achieving an even distribution by continuously swirling the plate before placing it back in the incubator. The supernatant was harvested at 42 h and 72 h post-transfection, combined and passed through a 0.45 μM filter before being aliquoted and stored at −80°C.

The following VOC S proteins were used to generate PVs. Mutations stated are compared to the Wuhan-Hu-1 isolate (MN908947.3). Corresponding Pango lineages are given in brackets:

— *D614G*. D614G;
— *Alpha* (*B.1.1.7*). Δ69–70, N501Y, D614G, P681H;
— *Beta* (*B.1.351*). K417N, E484K, N501Y, D614G, A701V;
— *Gamma* (*P.1*). K417T, E484K, N501Y, D614G, H655Y;
— *Delta* (*B.1.617.2*). L452R, T478K, D614G, P681R; and
— *Omicron* (*B.1.1.529*). A67V, Δ69-70, T95I, G142D, Δ143-145, N211I, Δ212, ins215EPE, G339D, S371L, S373P, S375F, K417N, N440K, G446S, S477N, T478K, E484A, Q493R, G496S, Q498R, N501Y, Y505H, T547K, D614G, H655Y, N679K, P681H, N764K, D796Y, N856K, Q954H, N969K, L981F.

#### (iv) Titration assays

Virus titration was carried out in 96-well plates by transduction of the firefly luciferase reporter into cells via the SARS-CoV-2 viral glycoprotein expressed by the PV. HEK293T/17 cells were transfected with ACE2 (NG_012575.3) and TMPRSS2 (AF270487.1), 24 h before viral infection. Negative controls consisted of cell-only wells. Positive controls were produced at the same time as the SARS-CoV-2 PV and comprised PV bearing the vesicular stomatitis virus G protein (VSV-G). VSV-G can use a wide variety of receptors to achieve viral entry and thus results in reliably high viral titres.

#### (v) Human embryonic kidney cells for use in experiments

HEK293T/17 cells for use in experiments were maintained in the same way as cells used for virus production, except in some cases where cells were transfected with murine ACE2 (NP_081562) or stably over-expressed human ACE2 and TMPRSS2 (NIBSC catalogue no. 101008).

#### (vi) Infectivity assays

Infectivity assays were set up in a similar manner for each of the cell types used. Cells were plated in a 96-well plate in order to reach a confluency of 2 × 10^5^ cells well^−1^ on the day of luminescence readout. In some experiments, LPS *Escherichia coli* 0111:B4 (Sigma, LPS25) was used to mimic heightened levels of inflammation [37]. Twenty-four hours after plating, LPS was added to cell cultures at a concentration of either 0.1, 1 or 10 μg ml^−1^ for 24 h. The following day the media containing LPS was removed from each well and replaced with 100 μl of media containing the PV. Each PV was serially diluted in duplicate onto each cell line, and the plates were then incubated for 24–48 h (5% CO_2_ at 37°C). After this, media was removed, and 30 μl of a solution containing Bright-Glo® (Promega) and serum-free media in equal parts was added to individual wells and left on a shaker for 2 min. After 5 min of incubation, the plate was read on a GloMax® Explorer Microplate Luminometer (Promega). Cells, alone, or incubated with delta-viral envelope protein (VEP), were used as negative controls. The titre (relative light units (RLU) ml^−1^) for the PV was calculated based on the raw RLU values recorded by the luminometer.

#### (vii) Data analysis

The resulting RLU scores from the luminometer were multiplied by the dilution factor applied to each well, and the mean from the 3 RLU ml^−1^ values for each PV variant was reported as the final number indicating the infectivity level. Plotting and statistical analysis of these values was conducted in R Studio, using linear mixed modelling as described in the text. Full statistical outputs are provided in the electronic supplementary material.

## 3. Results

### (a) Permissivity of HEK293T cells to SARS-CoV-2 pseudo-typed viruses varies depending on origin of S protein and levels of ACE2 and TMPRSS2 expressed, but not ACE2 species

**Figure 1.**
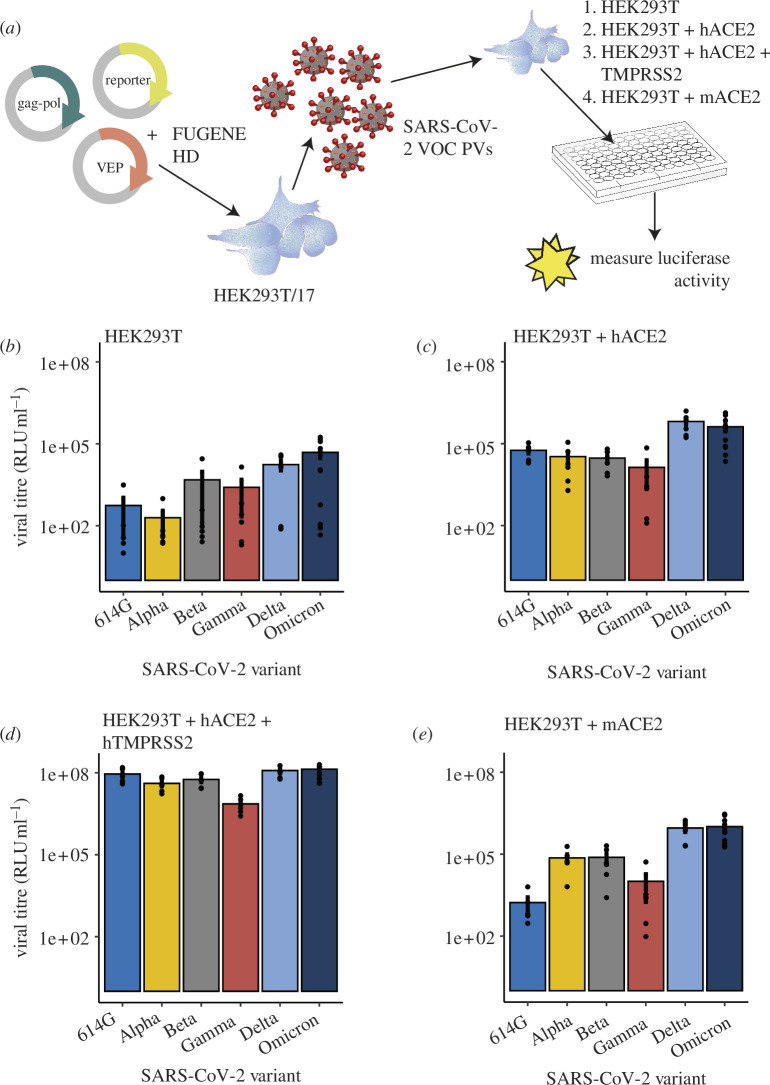
PV manufacture and infectivity of different HEK293T cell lines. (*a*) Schematic representation of PV production. Plasmids encoding the proteins required for PV assembly (gag-pol and VEPs for different SARS-CoV-2 VOCs) and a reporter protein (luciferase) were co-transfected into HEK293T/17 cells. Forty-eight and 72 h post-transfection, virus was collected and applied to different HEK293T cell lines, and infectivity was assessed using a luciferase assay. HEK293T cells image is from DataBase Center for Life Science [38]. Plate image is from Roy Granit [39]. (*b*–*e*) Infectivity of HEK293T cells expressing endogenous levels of ACE2 (*b*), or overexpressing human ACE2 (*c*), human ACE2 and human TMPRSS2 (*d*) or murine ACE2 (*e*) to PVs containing S proteins derived from different SARS-CoV-2 VOC were assessed. Bars represent mean RLU per ml ± s.e.m., with single dots representing individual samples (6–12 samples per condition, each from at least three biological repeats).

Pseudo-typed SARS-CoV-2 viruses were produced expressing VEPs for different SARS-CoV-2 VOC (figure 1*a*). Permissivity of HEK293T cells to SARS-CoV-2 PVs was significantly affected by both the VOC from which the S protein was derived and whether ACE2 and/or TMPRSS2 proteins were expressed in the cells (figure 1*b*,*e*; linear mixed model, with virus dilution as a random factor, main effect of PV variant: *p* = 3.8e−6; main effect of cell line: *p* < 2e−16). This was driven by increasing infectivity for the more recent SARS-CoV-2 variants (Delta and Omicron) as well as particularly high infectivity in the cells stably expressing human ACE2 and human TMPRSS2 (post hoc Tukey’s test versus HEK293T alone, HEK293T + hACE2 or HEK293T + mACE2: *p* < 1e−7).

**Figure 2.**
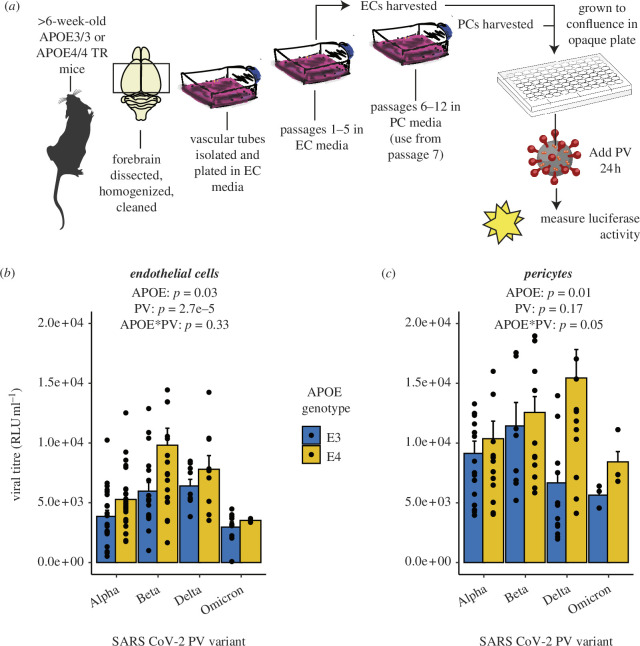
Infectivity of SARS-CoV-2 PVs is higher in mouse pericytes (PC) than endothelial cells (EC) and higher in cultures from APOE4-TR mice**.** (*a*) Schematic representation of experimental design (mouse and mouse brain images from Scidraw.io [42,43]). Cultures of endothelial cells and pericytes were prepared from forebrains of mice expressing APOE3 or APOE4 in place of murine APOE. Confluent cells were incubated with PVs expressing different SARS-CoV-2 variant S proteins. Infected cells express luciferase, which is detected using chemiluminescence. Luciferase activity (RLU ml^−1^) is shown for different PV variants applied to endothelial (*b*) and pericyte cultures (*c*). Data were analysed using a linear mixed model with virus dilution as a random factor. Figures show the results of post hoc tests of a further linear mixed model in which the analysis was split by cell type. Bars represent mean ± s.e.m., with single dots representing individual samples (6–54 samples per condition, from at least three experiments).

**Figure 3.**
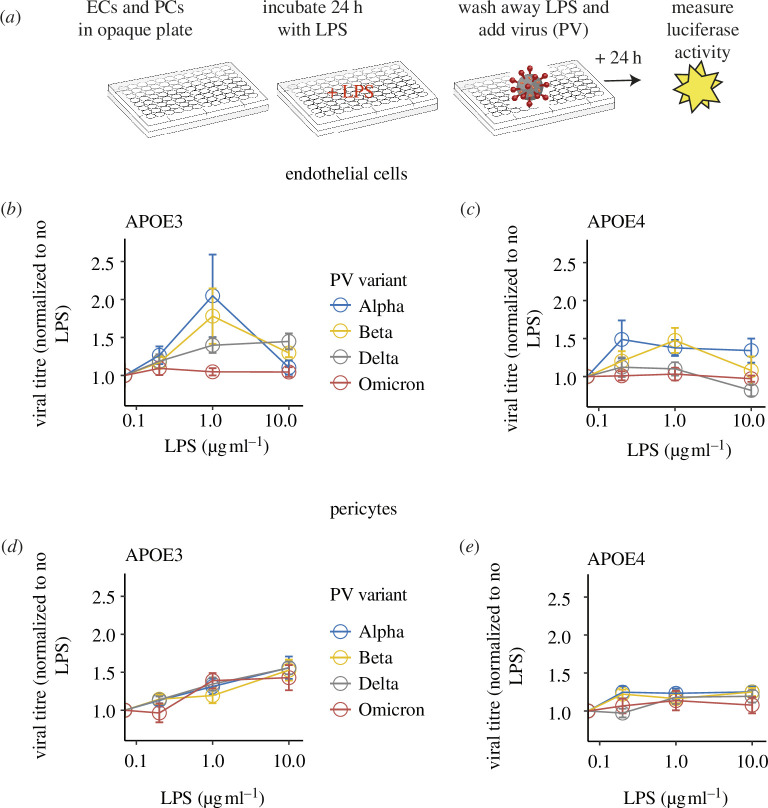
LPS increases vascular cell infectivity. (*a*) Schematic representation of experimental design. Endothelial cells (EC) and pericytes (PC) from APOE-TR mice were grown to confluence and then exposed to different doses of bacterial LPS for 24 h (0.1, 1.0 and 10.0 µg ml^−1^), before a media change to remove LPS and incubation for a further 24 h with the SARS-CoV-2 PV variants. PV infection was assayed from luciferase activity 24 h later. Dose responses for LPS effects on PV infection are shown for APOE3-TR endothelial cells (*b*), APOE4-TR endothelial cells (*c*), APOE3-TR pericytes (*d*) and APOE4-TR pericytes (*e*). Data represent mean ± s.e.m. from 6 to 27 samples per condition, each from at least three experiments. Individual data points are plotted in the electronic supplementary material, figure S2.

Early studies had reported poor binding of SARS-CoV-2 S protein to murine ACE2 [40] and therefore anticipated poor infectivity of murine cells. In our hands, and as previously reported [41], more recent SARS-CoV-2 variants show high infectivity in cells expressing murine ACE2 (figure 1*e*). There was no significant difference between infectivity of cells overexpressing human versus murine ACE2 (post hoc Tukey test, murine versus human ACE2-expressing cells; *p* = 1; figure 1*c*,*e*). Because murine ACE2 can permit access of more recent SARS-CoV-2 variants to the cell to a similar degree to human ACE2, in subsequent experiments we were able to use mouse cells to study the impact of genetic and inflammatory factors on the infectivity of SARS-CoV-2 PVs (figure 2) and human cells to study the impact of inflammation (figure 3).

### (b) Infectivity of SARS-CoV-2 pseudo-typed viruses is higher in APOE4 versus APOE3 cells and higher in pericytes than endothelial cells

Infectivity to SARS-CoV-2 PV was higher in endothelial (figure 2*b*) and pericyte cultures (figure 2*c*) from APOE4-TR mice compared to APOE3-TR mice (linear mixed model, with virus dilution as a random factor, main effect of genotype: *p* = 0.004). Infectivity was also higher in pericytes than endothelial cells (*p* = 3.7e−6) and differed across PV variants (*p* = 0.0013), although no individual pair-wise comparisons of different PV variants were significant (electronic supplementary material, table S1).

### (c) Prior inflammation, caused by lipopolysaccharide, increases pseudo-typed virus infection of murine pericytes but not endothelial cells

LPS increased PV infection of pericytes but not endothelial cells (linear mixed model with virus dilution as a random factor, cell type * LPS concentration interaction, *p* = 0.008; post hoc linear mixed models of each cell type separately: LPS effect on pericyte infectivity, *p* = 1.6e−8; on endothelial cells, *p* = 0.71; full statistical results are in the electronic supplementary material, table S2). Across all conditions, LPS trended towards increasing infectivity of SARS-CoV-2 PVs overall (LPS main effect: *p* = 0.07) and differed between PV variants (*p* = 0.03; although only Omicron and Alpha were significantly different in Tukey post hoc pairwise comparisons, *p* = 0.01). There was also a trend towards LPS increasing infectivity in APOE3-TR cells more than in APOE4-TR cells (APOE genotype * LPS interaction, *p* = 0.08, with LPS having a significant effect on APOE3-TR cells (*p* = 0.03) but not in APOE4 cells (*p* = 0.99)). Thus, prior LPS exposure increased infectivity of murine pericytes, particularly those of the APOE3 genotype.

### (d) Lipopolysaccharide alters SARS-CoV-2 pseudo-typed virus infectivity of HEK293T cells and human endothelial cells and pericytes

**Figure 4.**
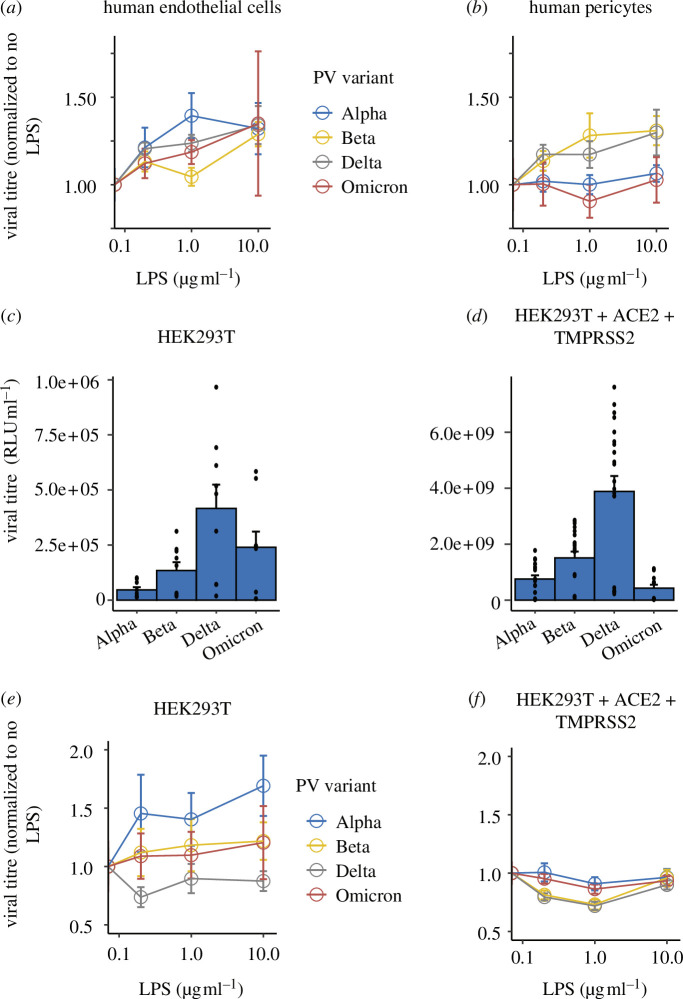
Effect of LPS on human vascular cell and HEK293T cell infectivity. (*a*) Dose responses for LPS effects on PV infection of human endothelial cells and (*b*) human pericytes. Data represent mean ± s.e.m. from 3 to 36 samples per condition, from at least three experiments. (*c*) Raw titres of infectivity of HEK293T cells and (*d*) HEK293T cells expressing human ACE2 and TMPRSS2 for different PVs in control conditions (no LPS). (*e*) Dose responses for LPS effects on PV infection of HEK293T and (*f*) HEK293T cells expressing human ACE2 and TMPRSS2. Data represent mean ± s.e.m. from 9 to 42 samples per condition, from at least three experiments. Individual data points are plotted in the electronic supplementary material, figure S2.

Human cardiac microvascular endothelial cells and human placental pericytes were both infected by SARS-CoV-2 variants, although variability between different batches was too great to be able to meaningfully compare cell-specific infectivity (electronic supplementary material, figure S3 and table S3). However, we could assess the impact of LPS on infectivity of both cell types, by normalizing data to baseline infectivity (no LPS). LPS significantly increased infectivity in both cell types (figure 4*a*,*b*; linear mixed model, main effect of LPS, *p* = 6e−4; post hoc analysis split by cell type: LPS effect on pericytes, *p* = 0.01, on endothelial cells, *p* = 0.02). Increasing LPS concentrations also differentially affected infectivity to different PVs, across the two cell types (cell * PV interaction, *p* = 0.017), with LPS affecting infectivity to Alpha and Omicron less than Delta and Beta in pericytes, but not in endothelial cells. Full statistical outputs are presented in the electronic supplementary material, table S4.

LPS also impacted the infectivity of HEK293T cells and HEK293T cells expressing ACE2 and TMPRSS2 to SARS-CoV-2 PVs (figure 4*d*–*f*; linear mixed model on data normalized to no LPS condition, with virus dilution as a random factor, main effect of LPS, *p* = 0.05), but this effect differed between HEK293T cells (figure 4*e*) and those transfected with ACE2 and TMPRSS2 (figure 4*f*; *p* = 0.0001) and between PV variant types (figure 4*e*,*f*; *p* = 0.0004). Notably, HEK293T cells expressing ACE2 and TMPRSS2, which had a higher baseline infectivity to SARS-CoV-2 PV variants, showed a decrease in infectivity after LPS treatment (figure 4*f*), whereas untransfected HEK293T cells showed a mixed response, with infectivity increasing to some variants and decreasing to others (figure 4*e*). Full statistical outputs are presented in the electronic supplementary material, table S5.

### (e) The effect of lipopolysaccharide is negatively correlated with baseline infectivity

**Figure 5.**
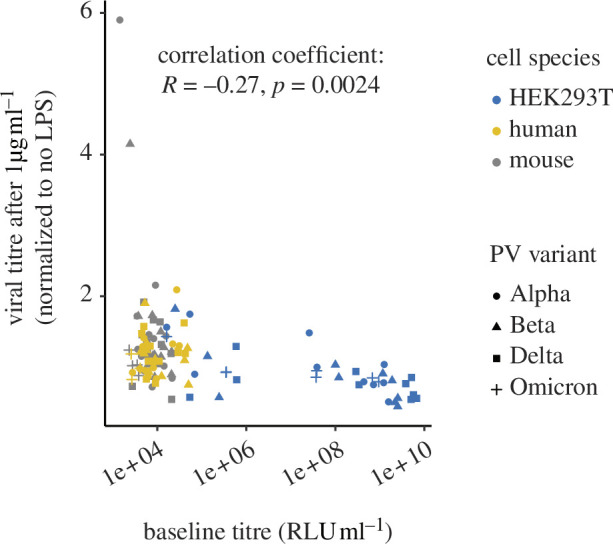
Response to LPS is negatively correlated with baseline infectivity. Average data for each experiment in figures 3 and 4 replotted showing the response to 1 µg ml^−1^ LPS against the infectivity in the same experiment in the absence of LPS. Pearson’s *R* was calculated from these values, and the two-tailed *p*-value was displayed.

We wondered if the different responses to LPS might relate to the baseline infectivity of cells, such that LPS increases the infectivity of cells with low baseline infectivity and reduces that of those with high baseline infectivity. Indeed, when we correlated the average responses for each condition across mouse and human vascular cells and different HEK293T cell variants, the LPS response was significantly negatively correlated with the baseline infectivity (figure 5).

## 4. Discussion

We found, as previously reported, that PVs expressing S protein from more recent SARS-CoV-2 variants (Alpha, Beta, Delta and Omicron) were able to more effectively use murine ACE2 as a cell receptor compared with the original Wuhan variant. These variants were able to infect both cultured murine and human endothelial cells and pericytes. PV infectivity was higher in murine cerebrovascular pericytes compared to endothelial cells and higher in cultures expressing APOE4 compared to APOE3. Increasing the inflammatory state of the cells by prior incubation with bacterial LPS increased infectivity of human and mouse pericytes and human endothelial cells. Thus, risk factors of severe COVID-19 infection (APOE4 genotype and prior inflammation) may be mediating some of their effects by increasing the ability of SARS-CoV-2 to infect vascular cells. Notably, the infectivity of the different cell types and the impact of APOE genotype and prior inflammation were different across PVs carrying S proteins from different VOCs, and the impact of prior inflammation appeared to be greatest in cells with the lowest baseline infectivity.

### (a) Cell-type specificity of SARS-CoV-2 infection

Endothelial dysfunction is an important feature of acute SARS-CoV-2 infection and long COVID [44–46]. Whether endothelial dysfunction occurs via effects of cytokines borne by the plasma [47] or by infection of endothelial cells themselves remains under debate. Some studies find low ACE2 expression and low infectivity of endothelial cells [48], while other studies find that endothelial cells can bind and internalize SARS-CoV-2 S protein in lung [49] and other tissues such as brain [50] and can cause degeneration of endothelial cells [27]. Pericyte expression of ACE2 has been reported to be much higher than that of endothelial cells, suggesting that vascular infection with SARS-CoV-2 might require breakdown of the blood–brain barrier to enable viral access to pericytes [51]. However, more recent work has suggested that SARS-CoV-2 can access cells independently of ACE2 via TMEM106B [28], which appears to be expressed in both endothelial cells and pericytes [23,29], although its levels at the plasma membrane of these cells are unclear. Here, we found lower infectivity to mouse endothelial cells when compared to pericytes, with infectivity of mouse endothelial cells only approximately 50% of that in pericytes. We were unable to compare levels of infectivity in human vascular cells owing to the high variability observed across supplied cell batches: in the first batch we received, pericytes showed higher infectivity, whereas the reverse was observed in the second batch of cells. Overall, these results suggest that endothelial cells and pericytes can both be infected by SARS-CoV-2 in humans and in mice, but that there is significant variability in the level of infection observed.

### (b) Multiple sources of variation in vascular cell infectivity

Some of the sources of variation in vascular infectivity remain unknown: we do not know what was different between our first and second batches of human endothelial cells and pericytes that produced such different levels of infectivity. However, other sources of variability are revealed by our data: APOE genotype, existing inflammatory state and the SARS-CoV-2 variant used impacted infectivity of both endothelial cells and pericytes, with effects often interacting to produce a wide range of different levels of observed infectivity. This variability, particularly that caused by the different VOC, might explain some of the mixed results in the literature regarding the infectivity of endothelial cells, depending on when studies using patient tissue were carried out (and therefore which was the dominant variant) or which recombinant S proteins were used.

### (c) Apolipoprotein E effects on vascular infectivity

APOE4 genotype has been linked to increased COVID-19 disease severity [30] and to increased cerebrovascular pathology, including microhaemorrhages [52]. It is not clear how this increased severity occurs, but one suggested mechanism is a direct interaction of APOE with ACE2, as APOE3 was found to bind and inhibit ACE2 internalization and virus entry to HEK293T cells more strongly than APOE4 [53]. In support of this idea, transcriptomic analysis suggests that APOE is expressed in human and mouse pericytes and endothelial cells, albeit at relatively low levels [23,54], and other studies support the occurrence of direct interactions between APOE and ACE2 [55]. However, this latter work reported no effect of APOE genotype on ACE2 binding and instead implicated increased APOE4-related disease severity to alterations in ACE2 expression and dysregulated renin-angiotensin signalling [55] or to a more general increased inflammatory state in APOE4 carriers [56,57]. Our results support a cellular rather than systemic level of impact of APOE genotype on SARS-CoV-2 effects, which could involve a direct interaction of APOE with ACE2 or proceed via a more general basal inflammatory state in APOE4 cells. The latter interpretation may be supported by our observation that increased LPS-induced inflammation increased vascular infectivity with a trend towards an increased impact on APOE3-TR versus APOE4-TR cells. This result is consistent with an overlap between the mechanisms of APOE4 and LPS-mediated increased infectivity, such that the APOE4-mediated effect occludes that of LPS treatment. Whatever the mechanism at the cellular level, *in vivo* the impact of increased infectivity in APOE4-TR cells would probably be exacerbated by the additional impact of APOE4-mediated reductions in blood–brain barrier integrity [58] that could allow better viral access to abluminal pericytes that are somewhat more infectable than the luminal endothelial cells.

### (d) Lipopolysaccharide effects on vascular infectivity

Systemic inflammation, owing to pre-existing conditions such as diabetes, coronary heart disease or obesity, is associated with increased morbidity and mortality after COVID-19 infection [59] and the development of long COVID [60]. The mechanism underlying this increased severity is not clear. Existing immune activation does not apparently mediate increased disease risk [61], but systemic inflammation increases the additional inflammatory response of COVID-19 infection as well as vascular damage and an increased risk of cytokine storm [60]. LPS may be directly involved in this process as inflammation-induced gut permeability may allow LPS leakage into the bloodstream, which can then bind SARS-CoV-2 and promote its internalization [62,63]. In our studies, LPS was not present at the same time as the SARS-CoV-2 PVs but was incubated with cells for 24 h and then removed before the addition of the PVs. This treatment increases the inflammatory state of the cells, as LPS stimulates toll-like receptor 4 to produce proinflammatory cytokines and type 1 interferons via complex intracellular pathways leading to parallel activation of transcription factors such as nucelar factor κ B (NFκB), interferon regulatory factors 3 and 5 (IRF3, IRF5) and activator protein 1 (AP1) [64]. The aspect of LPS signalling that leads to increased internalization of SARS-CoV-2 PVs is unclear, but NFκB is an interesting target, as its levels are also increased by APOE4 genotype [65] as well as conditions such as diabetes and obesity that increase the risk of severe COVID-19 disease [66]. Furthermore, inhibition of NFκB was found to decrease ACE2 expression in cultured human lung cells [67], suggesting that increases in NFκB could promote ACE2 expression and thus SARS-CoV-2 entry into cells. In support of this idea, our data showed an LPS-mediated enhancement of infectivity only in cells that did not already over-express ACE2, and a negative correlation between baseline infectivity and the increase in infectivity with LPS, suggesting that the potential to increase ACE2 expression might be necessary for the LPS effect.

### (e) Differences across SARS-CoV-2 variants

The SARS-CoV-2 PV variant often significantly impacted the infectivity of a given experiment, although there were often no significant differences between individual variants within an experiment. However, the Omicron PV tended to perhaps show lower infection rates and effects of LPS. As Omicron uses a different TMPRSS2-independent infection pathway [68], this may indicate that TMPRSS2 is involved in the LPS-mediated effect, but this would require direct testing in future work. Such variant-dependent vascular infectivity may contribute to the decreased rate of cardiovascular COVID-19 complications in Omicron patients [69,70] as well as the overall variability in reported results relating to vascular infectivity discussed above.

## 5. Conclusions

Human and mouse endothelial cells and pericytes can be infected with different variants of SARS-CoV-2 PVs. Vascular infectivity is modulated by the APOE genotype and increased inflammation, both risk factors for severe COVID-19 disease. This raises the possibility that the severity of COVID-19 depends in part on how effectively SARS-CoV-2 can access vascular tissues and thereby cause endothelial dysfunction and access to other tissues.

## Data Availability

The data presented are available at [71]. Electronic supplementary material is available online [72].
